# LONGITUDINAL QUALITATIVE RESULTS FROM STAFF IMPLEMENTING MONTESSORI DURING THE PANDEMIC

**DOI:** 10.1093/geroni/igae098.0081

**Published:** 2024-12-31

**Authors:** Michelle Hilgeman, Katherine Kennedy, Whitney Mills, A Snow, Teddy Bishop, Nathalie McIntosh, Kimberly Curyto

**Affiliations:** Tuscaloosa VA Medical Center, Tuscaloosa, Alabama, United States; VA Providence Healthcare System, Center of Innovation in Long-Term Services and Supports, Providence, Rhode Island, United States; VA Providence Health Care System, VA Providence Health Care System, Rhode Island, United States; Tuscaloosa VA Medical Center, Tuscaloosa, Alabama, United States; Tuscaloosa VA Medical Center, Tuscaloosa, Alabama, United States; Providence VA Medical Center, Providence, Rhode Island, United States; Western New York VA Healthcare System, Batavia, New York, United States

## Abstract

Promoting autonomy, dignity, and independence are core values of Montessori approaches to person-centered care in nursing homes. Yet infection control precautions restricted staff and resident freedoms during the COVID-19 pandemic. We describe longitudinal data from a stepped-wedge, randomized clinical trial examining implementation and effectiveness of Montessori approaches in eight Department of Veterans Affairs nursing homes (2021-2023). COVID-rates were calculated from electronic health records (EHRs) and graphed over 18 months around the intervention. Staff (N=168) participated in qualitative individual or group interviews at baseline, 3-, 6-, 9-, and 12-months. Normalization Process Theory informed questions and qualitative content analysis about the effects of COVID-19 on Montessori implementation and staff experiences. Transcripts were coded by members of the study team using NVivo. There was variability in COVID rates: most NHs concentrated below 1% of resident-days with COVID and occurred after 3-months of Montessori training/implementation, while two sites experienced peaks of 5% and 15%. Qualitative themes across implementation included: 1) Staff described fluctuating waves of COVID, changes in precaution protocols, and variable policy interpretations across sites to be associated with negative impact on residents and staff (isolation, loneliness, burden, burnout, low morale). 2) Staff identified creative adaptations (e.g. walkie-talkies) to implement Montessori within precaution protocols. 3) Staff reported that Montessori principals improved morale, mitigated negative effects of the pandemic and related precautions, and created a pathway for increased advocacy for person-centered care and resident wellbeing despite risks. Findings highlight the importance of reinforcing person-centered care in the midst of safety concerns during crisis.

